# Flexible taxonomic assignment of ambiguous sequencing reads

**DOI:** 10.1186/1471-2105-12-8

**Published:** 2011-01-07

**Authors:** José C Clemente, Jesper Jansson, Gabriel Valiente

**Affiliations:** 1 Center for Information Biology and DNA Databank of Japan, National Institute of Genetics, Yata 1111, Mishima 411-8540, Japan; 2 Department of Chemistry and Biochemistry, University of Colorado, Boulder, CO, USA; 3 Ochanomizu University, 2-1-1 Otsuka, Bunkyo-ku, Tokyo 112-8610, Japan; 4 Algorithms, Bioinformatics, Complexity and Formal Methods Research Group, Technical University of Catalonia, E-08034 Barcelona, Spain

## Abstract

**Background:**

To characterize the diversity of bacterial populations in metagenomic studies, sequencing reads need to be accurately assigned to taxonomic units in a given reference taxonomy. Reads that cannot be reliably assigned to a unique leaf in the taxonomy (*ambiguous reads*) are typically assigned to the lowest common ancestor of the set of species that match it. This introduces a potentially severe error in the estimation of bacteria present in the sample due to false positives, since all species in the subtree rooted at the ancestor are implicitly assigned to the read even though many of them may not match it.

**Results:**

We present a method that maps each read to a node in the taxonomy that minimizes a penalty score while balancing the relevance of precision and recall in the assignment through a parameter *q*. This mapping can be obtained in time linear in the number of matching sequences, because LCA queries to the reference taxonomy take constant time. When applied to six different metagenomic datasets, our algorithm produces different taxonomic distributions depending on whether coverage or precision is maximized. Including information on the quality of the reads reduces the number of unassigned reads but increases the number of ambiguous reads, stressing the relevance of our method. Finally, two measures of performance are described and results with a set of artificially generated datasets are discussed.

**Conclusions:**

The assignment strategy of sequencing reads introduced in this paper is a versatile and a quick method to study bacterial communities. The bacterial composition of the analyzed samples can vary significantly depending on how ambiguous reads are assigned depending on the value of the *q *parameter. Validation of our results in an artificial dataset confirm that a combination of values of *q *produces the most accurate results.

## Background

Microbes play a fundamental role as symbionts in the gut of mammals [1], and are strongly correlated with human health [2]. They also control some of the most important environmental processes, such as nitrogen fixation [3], and have been successfully used in the treatment of sewage [4] and to convert waste into usable fuels [5]. The importance of microbes is reflected in the large number of recent studies of bacterial communities in a variety of environments, including aquatic [6-11], soil [12-17], animal [18-23], and plant [24-29] habitats. The use of high-throughput sequencing technologies has greatly benefited the analysis of microbial populations [30], and different methodologies have been developed to characterize the diversity, richness, and similarity of bacterial communities [31-33]. Together with the introduction of new sequencing technologies, several challenges have emerged that need to be overcome to gain a better understanding of the diversity of bacteria that inhabit both the environment and ourselves [34].

Microbial communities are commonly characterized by mapping sequencing reads to a bacterial taxonomy based on the 16S rRNA gene. The effectiveness of this approach is not limited by the length of the read but by the choice of an adequate region of the 16S rRNA gene [35,36]. The structure of microbial communities has a high degree of variability, both in environmental [7] and gut samples [22]. In particular, human microbial communities differ greatly among individuals [23] and depending on the location of the body and time when the sample was taken [21]. Metabolic profiling has nevertheless shown that the functionality of communities is more conserved for particular environments [37], indicating that different species distributions can achieve a similar core functionality. Understanding the correlation between function and distribution of species therefore requires accurate measurements of both variables.

We have previously shown that a large proportion of reads in metagenomic studies can be assigned with equal significance to more than one species in the taxonomy [38]. The assignment of such *ambiguous *reads to the lowest common ancestor (LCA) of the matched species [39-42] introduces many false positives (leaves in the subtree rooted at the LCA that were not originally matched to the read), and thus we consider other possible nodes below the LCA to assign such reads. Implicitly, assignments at the LCA maximize the coverage but lower the accuracy, and we demonstrated that an assignment based on the *F*-measure, a combination of precision and recall, produces a significantly different distribution of taxonomic ranks to which reads are assigned.

In the absence of a reference taxonomy, the assignment of ambiguous reads is usually made by either mapping each read to the best BLAST hit in the reference sequences [43] or by using the reference sequences as a template for a multiple alignment of the reads, which defines pairwise similarities that are used to group the reads into clusters of related species [39,42,44]. In the absence of reference sequences, DNA composition can be used to group the reads into clusters of related species [45].

In this paper, we present a new method to assign ambiguous short reads to a node in the reference taxonomy minimizing a penalty score that generalizes our previous mapping based on the *F*-measure. Our algorithm is both fast and versatile, allowing a fine-grained assignment of reads closer to the LCA or the species depending on the value of a single parameter *q*. This parameter can be specified to have any value between 0 and 1, where setting *q *= 0 implies that each ambiguous read has an optimal assignment to a leaf, *q *= 1 always assigns to the LCA level, and *q *= 0.5 optimizes a combination of precision and recall. The use of this parameter provides the biologist with an intuitive tool to determine how to assign ambiguous reads, and results on six metagenomic datasets show the usefulness of our approach. The use of information on the quality of the sequencing reads results in a decrease in the number of unassigned reads but increases the number of ambiguous reads, thus making the assignment of such reads even more relevant. A method to validate our assignment algorithm is introduced and results for a set of artificial datasets are presented. Finally we discuss possible causes for the large proportion of ambiguous reads observed in these datasets.

## Methods

### Materials

We have initially studied six bacterial communities represented by 454 sequencing tags amplified from different 16S rRNA variable regions: a marine environment (V6 region) [7], the human gut (V3 and V6 regions) [20], the gut of lean and obese twins (V2 and V6 regions) [22], chicken gut (V6 region) [46], and rat gut (V4 region) [47]. The samples were between 50 and 329 bp in length and, for each of these communities, our algorithm assigned all the ambiguous sequencing reads at the best possible taxonomic rank, utilizing a reference bacterial taxonomy of 5,165 near-full-length type cultures of high quality [39], ranging in length between 1,202 and 1,780 bp, with a uniform scheme of seven taxonomic ranks (domain, phylum, class, order, family, genus, species). The taxonomy covers the whole spectrum of known bacteria, and the dominant phyla are Proteobacteria (1,925 species), Actinobacteria (1,285), Firmicutes (1,178), Bacteroidetes (355), and Tenericutes (160 species).

To test the effect of sequencing errors in the sequencing reads, we have also studied a dataset obtained from a bacterial community in the Priest Pot Lake [48], which included the quality scores for each read. Two taxonomies were used for this experiment, one based on the 5,165 high-quality type cultures only and the other one using all 322,864 16S rRNA sequences found in the Ribosomal Database Project [39].

Validation of our assignment algorithm was performed using artificial datasets generated with MetaSim [49], as follows. A first dataset was created importing 5,148 16S rRNA sequences from the taxonomy into MetaSim, and creating a dataset of short sequencing reads with the following parameters: 454 error model; 5,000,000 of reads per run; normal DNA clone size distribution with mean 500 and standard deviation 20; and expected read length 100, 150, and 200. Reads of less than 75% the expected length were discarded. A second dataset was created by extracting first 300 base pairs roughly corresponding to hypervariable regions V1 and V2 in each of the 5,148 rRNA sequences, and then importing those subsequences into MetaSim to generate a new set of short reads using the same set of parameters as before.

### Taxonomic Assignment Method

In this section, we introduce a new method for accurately assigning a sequencing read *R_i _to a single node *in a fixed reference taxonomy tree *T *with a leaf set *L*. We assume that each leaf in *L *has an associated known sequence. The input is a set *R *of sequencing reads and a positive integer *k*. For each *R_i _*∈ *R*, there is a subset *M_i _*⊆ *L *of leaves whose sequences contain a substring with at most *k *mismatches to *R_i_*; these leaves are referred to as *hits *or *matches *below. The goal is to output, for each *R_i _*∈ *R *with |*M_i_*| ≥ 1, one node in *T *which represents all of *M_i _*in a "good" way.

For any *R_i _*∈ *R*, if |*M*_*i*_| = 1 then *R_i _*can be trivially assigned to a unique leaf. However, if |*M*_*i*_| ≥ 2 then *R_i _*is called an *ambiguous read *and it is not immediately obvious how to optimally assign *Ri *to one node in *T*.

For this purpose, we let the user specify a value in the interval [0, 1] for a new parameter *q*. Intuitively, setting a low value of *q *means that ambiguous reads will be assigned to nodes near the leaves, while a high value of *q *means that assignments near the LCA level are preferred. Our approach for taxonomic assignment of reads is as follows.

1. Apply a read mapping tool, such as GEM [50] for instance, to *R *to compute the set of hits *M_i _*for every *R_i _*∈ *R*.

2. Let the user specify a value in the interval [0, 1] for the parameter *q*.

3. For each *R_i _*∈ *R*:

(a) If |*M*_*i*_| = 0 then output *null*.

(b) If |*M*_*i*_| = 1 then output the leaf in *M_i_*.

(c) Else, output all nodes *j *of *T *that have the smallest possible *penalty score PS*_*i*,*j *_with respect to *q*.

In the following sections we give the formal definition of the penalty score *PS_i,j _*and study some of its properties. Then, we consider how to implement Step 3c of our method efficiently, that is, how to compute the *PS_i,j _*values quickly.

#### Definition of the *Penalty Score PS_i,j_*

Let *T *be a (fixed) rooted tree with a leaf set *L*, let *M_i _*⊆ *L*, and let *q *be a real number in the interval 0[1].

We need some additional notation. Let *Ti *be the subtree of *T *that is rooted at the LCA of *M_i_*. For every node *j *in *T_i_*, define:

• *T_i,j _*= The subtree of tree *T_i _*rooted at node *j*.

• True positives: *TP_i,j _*= Leaves in *T_i,j _*that belong to *M_i_*.

• False positives: *FP_i,j _*= Leaves in *T_i,j _*that do not belong to *M_i_*.

• True negatives: *TN_i,j _*= Leaves in *T_i_\T_i,j _*that do not belong to *M_i_*.

• False negatives: *FN_i,j _*= Leaves in *T_i_\T_i,j _*that belong to *M_i_*.

**Figure 1:**
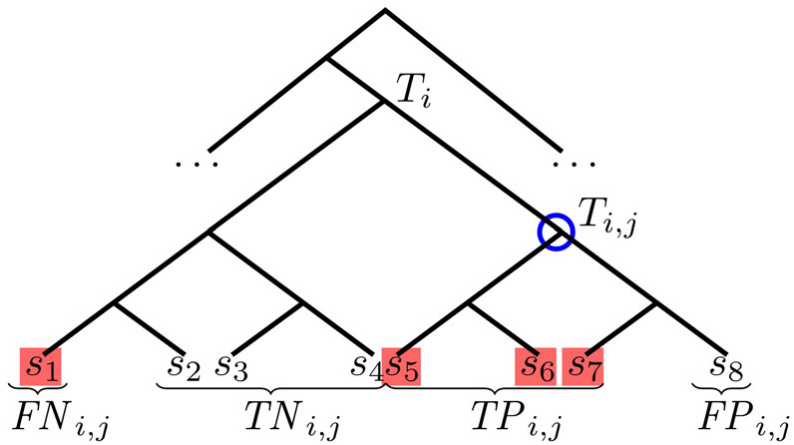
**Sample Taxonomic Assignment of an Ambiguous Read**. Assigning read *R_i _*to the *j*th node of *T_i _*partitions the leaves of *T_i _*into true positives, false positives, true negatives, and false negatives. In this example, the hits are *M_i _*= {*s*_1_, *s*_5_, *s*_6_, *s*_7_}. If we let *j *be the blue circled node, we obtain *TP_i,j _*= {*s*_5_, *s*_6_, *s*_7_}, *FP_i,j _*= {*s*_8_}, *TN_i,j _*= {*s*_2_, *s*_3_, *s*_4_}, *FN_i,j _*= {*s*_1_}. True hit *H *= *s*_5 _(defined in Section "Validation: Performance in ROC Space").

See Figure 1 for an example. Note that for each node *j *in *T_i_*, the leaves of *T_i _*are partitioned into four disjoint subsets *TP_i,j_*, *FP_i,j_*, *TN_i,j_*, and *FN_i,j_*. The interpretation of this is that in case the node *j *is selected to be the representive for a read *R_i _*whose hits are *M_i_*, then each leaf in *T_i _*will either be a true positive, a false positive, a true negative, or a false negative depending on whether or not it lies in the subtree rooted at *j *and if it is one of the hits.

Finally, we define the *penalty score with respect to q *for every node *j *in *T_i _*by the following formula:

(1) PSi,j=q⋅(|FNi,j|/|TPi,j|)+(1−q)⋅(|FPi,j|/|TPi,j|)

For every node *j *of *T *that does not belong to *T_i_*, we define *PS_i,j _*= ∞. In case |*TP_i,j_*| = 0 then we also define *PS_i,j _*= ∞.

#### Different Values of q

Recall that our method for taxonomic assignment assigns each ambiguous read *R_i _*to a node *j *that minimizes the value of *PS_i,j _*for the particular value of *q *specified by the user. We now study how varying the value of *q *affects the resulting taxonomic assignments.

First, it is easy to see that selecting *q *= 0 implies that a read *R_i _*may have several different optimal assignments, but there always exists an optimal assignment of *R_i _*to a leaf in *T_i _*since then |*FP_i,j_*| (and hence, *PS_i,j_*) will be zero. On the other hand, *q *= 1 always assigns each *R_i _*to the unique LCA of *M_i _*because this gives |*FN_i,j_*| = 0 and *PS_i,j _*= 0. Thus, the special case *q *= 1 corresponds to the currently commonly used methods for assigning ambiguous reads [39-42].

Furthermore, we observe that *PS_i,j _*is a generalization of the mapping based on the *F*-measure that we previously introduced in [38]. If the precision of classifying read *R_i _*into *T_j _*is *P_i,j _*= |*TP_i,j_|/*(|*TP_i,j_*| + |*FP_i,j_*|) and the recall is *R_i,j _*= |*TP_i,j_|/*(|*TP_i,j_*| + |*FN_i,j_*|), then the *F*-measure is *F_i,j _*= 2*P_i,j_R_i,j_/*(*P_i,j _*+ *R_i,j_*) = 2|*TP_i,j_|/*(|*FN_i,j_*| + |*FP_i,j_*| + 2|*TP_i,j_*|)). This yields:

**Lemma 1**. *Any node m that minimizes the penalty score for q *= 0.5 *also maximizes the F-measure*.

*Proof*.

$$\begin{array}{l} \arg\underset{m}{\min}(|FN_{i,m}|/|TP_{i,m}|+|FP_{i,m}|/|TP_{i,m}|)\\ =\arg\underset{m}{\min}((|FN_{i,m}|+|FP_{i,m}|)/|TP_{i,m}|)\\ =\arg\underset{m}{\min}((|FN_{i,m}|+|FP_{i,m}|)/2|TP_{i,m}|)\\ =\arg\underset{m}{\min}(((|FN_{i,m}|+|FP_{i,m}|)/2|TP_{i,m}|)+1)\\ =\arg\underset{m}{\min}((|FN_{i,m}|+|FP_{i,m}|+2|TP_{i,m}|)/2|TP_{i,m}|)\\ =\arg\underset{m}{\max}(2|TP_{i,m}|/(|FN_{i,m}|+|FP_{i,m}|+2|TP_{i,m}|))\\ =\arg\underset{m}{\max}(2P_{i,m}R_{i,m}/(P_{i,m}+R_{i,m})). \end{array}$$

To summarize, the parameter *q *directly influences where in the reference taxonomy ambiguous reads will be assigned to. The user can adjust *q *to obtain representatives for ambiguous reads at the leaf level (*q *= 0), the LCA level (*q *= 1), or somewhere in-between (0 <*q *< 1). Interestingly, *q *= 0.5 is equivalent to maximizing the *F*-measure, which optimizes a combination of precision and recall. The distribution of taxonomic ranks resulting from setting various values of *q *in [0, 1] is further investigated for some real datasets in the "Results" Section.

#### Computation of the Penalty Scores PS_i,j_

Here, we focus on how to compute the penalty scores *PS_i,j _*in Step 3c of our method efficiently. For any tree *T*, let |*T*| denote the number of nodes in *T*. As before, let *M_i _*be a set of hits in the reference taxonomy tree *T *and let *T_i _*be the subtree of *T *that is rooted at the LCA of *M_i_*. We may assume that |*M_i_| ≥ *2. Below, we first describe a simple method to obtain *PS_i,j _*for every node *j *in *T_i _*in *O*(|*T_i_*|) total time (Theorem 1). Then, we show that if *O*(|*T_j_*|) time preprocessing of *T *is allowed, we can reduce the time complexity to obtain *PS_i,j _*for every node *j *in *T_i _*in *O*(|*M_i_*|) total time (Theorem 2). (The preprocessing of *T *will be done *once*, before any other computations in our taxonomic assignment method.) This modification gives a significant speedup in case *R *contains many reads that induce small sets of hits whose LCA are located at high taxonomic ranks in *T*.

For every node *j *in *T_i_*, define *T_i,j _*as the subtree of tree *T_i _*rooted at *j*. The set of all leaves in *T_i _*is denoted by *L_i_*, and *N_i _*is the set of all leaves in *T_i _*that do not belong to *M_i _*(hence, *L_i _*= *M_i _*∪ *N_i_*). Similarly, the set of all leaves in *T_i,j _*that belong to *M_i _*is denoted by *M_i, j_*, *N_i,j _*is the set of all leaves in *T_i,j _*that do not belong to *M_i, j_*, and *L_i,j _*= *M_i,j _*∪ *N_i, j_*. Using this notation, we can write the previously defined *TP_i, j_*, *FP_i, j_*, *TN_i, j_*, and *FN_i,j _*as:

• True positives: *TP_i,j _*= *M_i, j_*

• False positives: *FP_i,j _*= *N_i, j_*

• True negatives: *TN_i,j _*= *N_i _\N_i, j_*

• False negatives: *FN_i,j _*= *M_i _\M_i, j_*

Next, we rewrite the formula for the penalty score in terms of |*M_i_*|, |*M_i, j_*|, and |*N_i, j_*| as:

(2) $$\begin{array}{c} PS_{i,j}=\frac{q\cdot |FN_{i,j}|+(1-q)\cdot |FP_{i,j}|}{\left|TP_{i,j}\right|}\\ =\frac{q\cdot (|M_{i}|-|M_{i,j}|)+(1-q)\cdot |N_{i,j}|}{\left|M_{i,j}\right|} \end{array}$$

Since *M_i _*is given, the value of |*M_i_*| is fixed. For any node *j *in *T_i_*, the values of |*M_i, j_*| and |*N_i, j_*| may be expressed recursively as follows:

• If *j *is a leaf in *T_i _*and *j *∉ *M_i_*: Then |*M_i, j_*| = 1, |*N_i, j_*| = 0, and |*L_i, j_*| = 1.

• If *j *is a leaf in *T_i _*and *j *∉ *M_i_*: Then |*M_i, j_*| = 0, |*N_i, j_*| = 1, and |*L_i, j_| *= 1.

• If *j *is an internal node in *T_i_*: Then |*M_i,j_*| = ∑*_j'_*|*M_i,j'_*| and |*L_i,j_*| = ∑*_j'_*|*L_i,j'_*|, where *j' *ranges over the children of *j *in *T_i_*, and |*N_i, j_| *= |*L_i, j_| - |M_i, j_*|.

Hence, the values of *PS_i,j _*for all nodes in *T_i _*can be obtained by two traversals of *T_i_*: a (partial) bottom-up traversal [51,52] to identify the root of the subtree *T_i _*of *T *(start at the leaves belonging to *M_i _*and end when a node that is an ancestor of all leaves from *M_i _*is reached; which can be determined by storing, for each node, the number of descendent leaves from *M_i_*, because the first node in the bottom-up traversal that is an ancestor of all leaves from *M_i _*has exactly |*M_i_| *descendent leaves from *M_i_*) followed by a top-down traversal to identify the subtree *T_i _*of *T *while computing |*M_i, j_*|, |*N_i, j_*|, |*L_i, j_*|, and *PS_i,j _*for all nodes in *T_i _*by applying the above relations. There are *O*(|*T_i_*|) nodes in *T_i_*, and so we have:

**Theorem 1**. *We can find a node j in T that minimizes the value of PS_i,j _for any M_i _*⊆ *L in O*(|*T_i_*|) *time*.

Next, we present an alternative method that improves the time complexity stated in Theorem 1 if preprocessing of the reference taxonomy tree *T *is allowed.

We start by explaining how to preprocess *T*. Fix an arbitrary left-to-right ordering of the nodes in *T *and perform a left-to-right postorder traversal of *T *in *O*(|*T*|) time while enumerating the nodes from 1 to |*T| *in accordance with the order in which they are first visited. Associate each node *j *with its number and, moreover, keep track of the smallest numbered leaf in the subtree rooted at *j *and denote it by *m*(*j*). Subsequently, for any two nodes *j*, *j' *in *T*, it holds that *j *is a proper ancestor of *j' *if and only if *m*(*j*) ≤ *m*(*j'*) ≤ *j' *<*j*, and this condition can be checked in *O*(1) time. (The intervals [*m*(*j*), *j*] induced by nodes in *T *therefore exhibit a nested structure that will be utilized below.) Next, apply the *O*(|*T*|)-time preprocessing of [53,54] to *T *so that the LCA of any pair of specified leaves from *T *can be obtained in *O*(1) time, unless the height of *T *is bounded by a constant (usual taxonomical classifications as kingdom-phylum-class-order-family-genus-species have height 8, and the NCBI taxonomy [55] has a few more levels to account for finer distinctions), in which case the LCA of any pair of specified leaves from *T *can readily be obtained in *O*(1) time, without any preprocessing [52]. Lastly, do a *O*(|*T*|)-time bottom-up traversal of *T *to compute and store the number of leaves |*L_i_| *in the subtree rooted at each node *i *in *T *.

Now, suppose the preprocessing has been taken care of and we are given a set *M_i _*⊆ *L *of hits. Any node *j *in *T_i _*is called *relevant *if it is equal to a leaf in *M_i _*or equal to the LCA of two or more leaves in *M_i_*. We have:

**Lemma 2**. *For each node j in T_i_*, *there exists a relevant node j' such that PS_i, j' _**≤ **PS_i, j_*.

*Proof*. Suppose that *j *is a node in *T_i _*that is not relevant. Let *j' *be the LCA of the leaves in *M_i, j_*. Clearly, *j' *is relevant and, furthermore, |*M_i, j_| *= |*M_i, j'_| *while |*N_i, j_| > |N_i, j'_| *since *T_i, j' _*is a subtree of *T_i, j_*. It follows that $PS_{i,j^{\prime }}-PS_{i,j}=(1-q)\cdot \frac{\left|N_{i,j^{\prime }}\right|}{\left|M_{i,j^{\prime }}\right|}-(1-q)\cdot \frac{\left|N_{i,j}\right|}{\left|M_{i,j}\right|}=(1-q)\cdot \frac{\left|N_{i,j^{\prime }}|-|N_{i,j}\right|}{\left|M_{i,j}\right|}\leq 0$.

Lemma 2 implies that *PS_i,j _*only needs to be computed for nodes in *T_i _*that are relevant. Define the *topological restriction of T_i _to M_i_*, denoted by *T_i _*|| *M_i_*, as the tree obtained by deleting from *T_i _*all nodes that are not on a path from the root to a leaf in *M_i _*along with their incident edges, and then contracting every edge between a node having just one child and its child. Then, the nodes of *T_i _*|| *M_i _*are precisely the relevant nodes in *T_i_*. Observe that *T_i _*|| *M_i _*contains *O*(|*M_i_*|) nodes.

To construct *T_i _*|| *M_i _*for any specified *M_i _*⊆ *L *in *O*(|*M_i_*|) time, proceed as follows. Sort the leaves in *M_i _*in *O*(|*M_i_*|) time according to their left-to-right postordering numbers by a radix sort and write $M_{i}=\{\ell_{1},\ell_{2},\ldots ,\ell_{\left|M_{i}\right|}\}$ with $\ell_{1}<\ell_{2}<\cdots <\ell_{\left|M_{i}\right|}$. For *x *∈ {1, 2, ..., |*M_i_| *, perform an *O*(1)-time LCA query on the pair (ℓ*_x_*, ℓ_*x*+1_) and let *k_x _*be the answer. The set $U=M_{i}\int \{k_{1},k_{2},\ldots ,k_{|M_{i}|-1}\}$ then gives the set of nodes in *T_i _*|| *M_i_*. To obtain the edges of *T_i _*|| *M_i_*, first use *O*(|*M_i_*|) time to perform a radix sort on the set of ordered pairs {(*m*(*j*), *j*): *j *∈ *U*} in *non-decreasing order *for the first coordinate and *decreasing order *for the second coordinate so that in the resulting ordering, for any *j*, *j'*∈ *U*, it holds that (*m*(*j*), *j*) < (*m*(*j'*), *j'*) if and only if either (1) *m*(*j*) <*m*(*j'*), or (2) *m*(*j*) = *m*(*j'*) and *j *>*j'*. Thus, whenever a node *j *is a proper ancestor of a node *j'*, the pair (*m*(*j*), *j*) appears somewhere before (*m*(*j'*), *j'*) in the sorted list. Then, it is straightforward to recover the edges of *T_i _*|| *M_i _*in *O*(|*M_i_*|) time by traversing the sorted list of pairs while using a stack to store all proper ancestors of the currently considered node (recall that it takes *O*(1) time to check the condition *m*(*j*) ≤ *m*(*j'*) ≤ *j' < j *for any node *j' *in the list and any element *j *on the top of the stack).

Finally, the values of *PS_i,j _*for all relevant nodes can be obtained by a bottom-up traversal of *T_i _*|| *M_i_*. There are *O*(|*M_i_*|) relevant nodes, and so we can compute the values of *PS_i,j _*for all relevant nodes *j *in Step 3c according to formula (2) using *O*(|*M_i_*|) time. To do this, note that if *j *is an internal node in *T_i _*|| *M_i _*then |*M_i_*, *j*| = ∑*_j' _*|*M_i_, j'*|, where *j' *ranges over the children of *j *in *T_i _*|| *M_i_*, and |*N_i_*,*j| *= |*L_i_*,*j*| - |*M_i_*,*j*|, where |*L_i_*,*j*| = |*L_j_*| has been precomputed. In total:

**Theorem 2**. *After O*(|*T*|) *time preprocessing*, *we can find a node j in T that minimizes the value of PS_i,j _for any M_i _*⊆ *L in O*(|*M_i_*|) *time*.

### Validation: Performance in ROC Space

Each read found in a metagenomic dataset must have originated from a unique original 16S rRNA sequence but, due to sequencing errors and incomplete taxonomic information, a significant percentage of the reads end up being assigned at higher taxonomic levels. Using an artificial metagenomic dataset, we can know *a priori *the original sequence and therefore measure how accurately our algorithm classifies ambiguous reads. We used MetaSim to generate an artificial set of sequencing reads *R *with a 454 error model, and where each read *R_i _*is derived from a randomly selected full-length 16S rRNA gene sequence annotated in the taxonomy, denoted by *H_i_*. Because of the errors introduced in *R_i _*by the simulation, a search for the most similar full-length sequences to the read produces a set of hits, *M_i_*, among which the true hit is usually found. The tree *T_i _*rooted at the LCA of *M_i _*can therefore include three kinds of leaves: the true hit *H_i_*, not true but ambiguous hits *M_i _\*{*H_i_*}, and false hits *L_i _\M_i _*= *N_i_*.

When using our *q*-assignment schema, the sets *M_i_*, *N_i_*, *M_i,j_*, and *N_i,j _*are defined with respect to the set of plausible hits but without knowledge of the true hit *H_i_*, as shown in Figure 1. Our objective here is to measure how different values of *q *perform in including *H_i _*among the selected leaves. Assignments to the LCA will in most cases include *H_i _*but the precision will be very poor if the size of *T_i _*is large, while lower values of *q *can increase the precision at the risk of excluding *H_i_*.

A common measure of performance for binary classifiers is the area under the ROC curve [56]. For a given read *R_i _*and a particular value of *q*, let us define the true positive rate with respect to the true hit *H_i _*as $TPR_{H_{i}}=|TP_{H_{i}}|/(|TP_{H_{i}}|+|FN_{H_{i}}|)$ when *H_i _*∈ *T_i _*and 0 otherwise (if *H_i _*∉ *T_i _*both $TP_{H_{i}}$ and $FN_{H_{i}}$ would be empty). Notice that when previously calculating the assignment we used the sets *TP*, *FP*, *TN*, and *FN *with respect to *M*_*i*_, while here we calculate $TP_{H_{i}},FP_{H_{i}},TN_{H_{i}}$, and $FN_{H_{i}}$ taking into account *H_i _*only. In a similar way we define $FPR_{H_{i}}=|FP_{H_{i}}|/(|FP_{H_{i}}|+|TN_{H_{i}}|)$. However, $TPR_{H_{i}}$ can only be 1 (when *H_i _*is in the subtree rooted at the node to which *R_i _*was assigned) or 0 (when *H_i _*is not in the subtree) and therefore, plotting *TPR *versus *FPR *would result in degenerated ROC curves.

We need to define $TP_{H_{i}},FP_{H_{i}},TN_{H_{i}},FN_{H_{i}}$ as sets of leaves. That is, $|TP_{H_{i}}|=1$ if *H_i _*∈ *T_i_*, and $|TP_{H_{i}}|=0$ otherwise. Then, $\left|TP_{H_{i}}|=\{H_{i}\right\}$ if *H_i _*∈ *T_i_*, and $TP_{H_{i}}=\emptyset$ otherwise.

**Figure 2:**
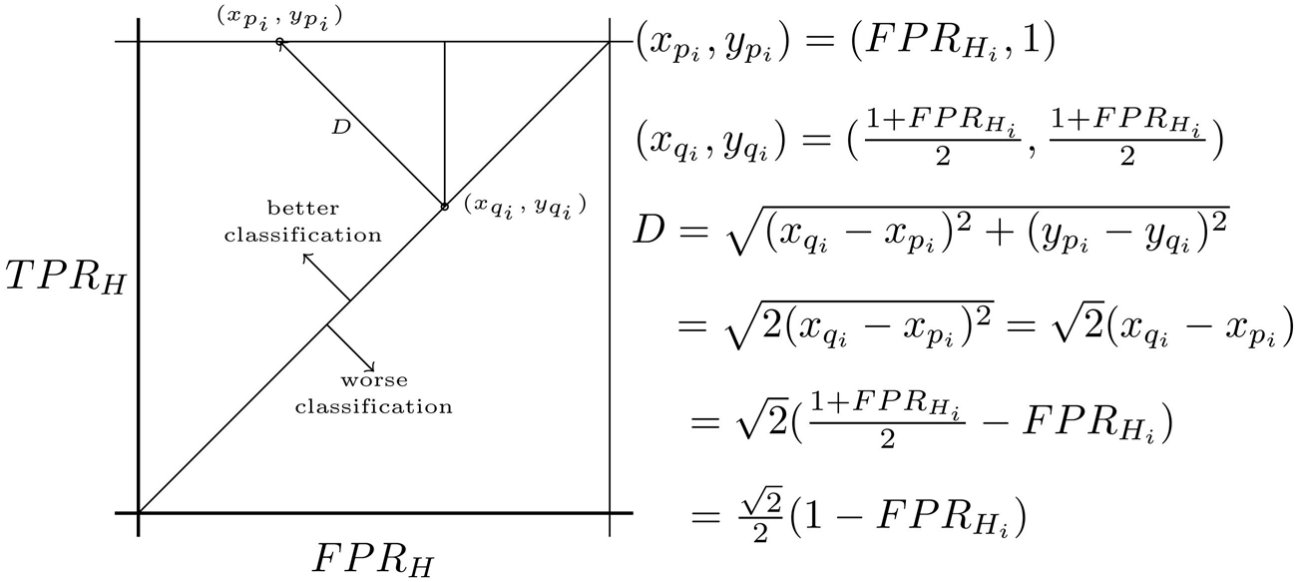
**Validation of Results in ROC Space**. Distance in ROC space to the diagonal *TPR_H _*= *FPR_H_*. Points above the diagonal represent good predictions, the larger the distance the better.

Let us define $p_{i}=(FPR_{H_{i}},TRP_{H_{i}})$ as the point in ROC space that represents read *R_i_*. Points above the diagonal *FPR *= *TPR *have good predictive power, points below it are poor classification results, and points on the diagonal have no predictive power; that is, they are a random guess. The distance of *p_i _*to the diagonal is denoted by *D_i_*, and corresponds to the distance between *p_i _*and the point of intersection between the diagonal and the perpendicular that goes through pi. As shown in Figure 2*D_i _*equals $\sqrt{2}(1-FPR_{H_{i}})/2$, and we can define the goodness *G_q _*of an assignment for a particular value of q as the sum of distances to the diagonal for all reads, where distances are negative if a point lies below the diagonal:

(3) $$G_{q}=\sum_{i}D_{i}=\sum_{i}\frac{\sqrt{2}}{2}(1-FPR_{H_{i}})$$

The value of *q *that maximizes this sum will be the one with highest predictive power. This measure will be called the q metric of performance. Notice that the distance *D_i _*differs both in sign and in value depending on whether Hi is in the subtree *T_i,j _*or not. We will define $D_{H\in T_{i,j}}$ as the distance for read *R_i _*when Hi is in the subtree and $D_{H\notin T_{i,j}}$ when it is not:

$$\begin{array}{c} D_{H_{i}\in T_{i,j}}=\frac{\sqrt{2}}{2}(1-FPR_{H\in T_{i,j},i})\\ =\frac{\sqrt{2}}{2}(1-\frac{|L_{i,j}|-1}{|L_{i}|-1})\\ =\frac{\sqrt{2}}{2}\frac{\left|L_{i}|-|L_{i,j}\right|}{|L_{i}|-1}\\ D_{H_{i}\notin T_{i,j}}=-\frac{\sqrt{2}}{2}(1-FPR_{H\notin T_{i,j},i})\\ =-\frac{\sqrt{2}}{2}(1-\frac{\left|L_{i,j}\right|}{|L_{i}|-1})\\ =-\frac{\sqrt{2}}{2}\frac{|L_{i}|-|L_{i,j}|-1}{|L_{i}|-1} \end{array}$$

For a given value of *q*, our assignment produces two distinct subsets of reads: those that have been assigned to a node among whose descendents the true hit *H_i _*can be found, and those that do not have *H_i _*as a descendent. The goodness of an assignment for a value of *q *can then be rewritten as:

$$\begin{array}{c} G_{q}=\sum_{i}D_{H_{i}\in T_{i,j}}+\sum_{k}D_{H_{i}\notin T_{k,j}}\\ =\frac{\sqrt{2}}{2}\left(\sum_{i}\frac{\left|L_{i}|-|L_{i,j}\right|}{|L_{i}|-1}-\sum_{k}\frac{|L_{k}|-|L_{k,j}|-1}{|L_{k}|-1}\right) \end{array}$$

Alternatively, and instead of assigning ambiguous reads based on the unique *q *value that maximizes *G_q_*, we can look for the assignment that maximizes the expected distance *E*(*D_i_*) for each read, so our mapping would use a combination of different *q *values depending on the particular read being considered. Let us assume read *R_i _*can be assigned to nodes *n*_1_, ..., *n_n _*for each of the *q *values *q*_1_, ..., *q_n_*. The probability of Hi being among the leaves of the subtree *T_i,j _*rooted at *n_j _*is *p *= *M_i,j_/M_i_*, and that of not being included is 1 - *p *= (*M_i _*-*M_i,j_*) = *M_i_*. The expected distance for a read Ri mapped to a node j for a given q is:

$$\begin{array}{c} E(D_{i,j})=pD_{H_{i}\in T_{i,j}}+(1-p)D_{H_{i}\notin T_{i,j}}\\ =\frac{\left|M_{i,j}\right|}{\left|M_{i}\right|}\frac{\sqrt{2}}{2}\frac{\left|L_{i}|-|L_{i,j}\right|}{|L_{i}|-1}\\ \;-\frac{\left|M_{i}|-|M_{i,j}\right|}{\left|M_{i}\right|}\frac{\sqrt{2}}{2}\frac{|L_{k}|-|L_{k,j}|-1}{|L_{k}|-1}\\ =\frac{\sqrt{2}}{2}(\frac{\left|M_{i,j}|(2|L_{i}|-2|L_{i,j}|-1\right)}{\left|M_{i}|(|L_{i}|-1\right)}\\ \;+\frac{\left|M_{i}|(|L_{i,j}|-|L_{i}|+1\right)}{\left|M_{i}|(|L_{i}|-1\right)}) \end{array}$$

We will call this measure of performance the *expected distance metric*. The value of *q *(and the corresponding node) that maximizes the expected distance for *R_i _*is chosen as the most appropriate if the distance is positive, otherwise the assignment to the LCA is preferred. It can be easily seen that assignments to the LCA when the true hit *H_i _*is among its leaves have expected distance 0, since *M_i_*,*j *= *M_i_*. Lemma 3 shows that the true distance for assignments to the LCA is always zero and, therefore, such assignments have no predictive power.

**Lemma 3**. *Assignments to the LCA have no predictive power when the true hit H_i _is among its leaves*.

*Proof*. Since the true hit *H_i _*is among the leaves of the subtree rooted at the LCA, the $TPR_{H_{i}}$ is 1. The $FPR_{H_{i}}$ is $\left|FP_{H_{i}}|/(|FP_{H_{i}}|+|TN_{H_{i}}|\right)$ and, since assigning to the LCA selects all possible leaves, $TN_{H_{i}}=\emptyset$ and $FPR_{H_{i}}=1$. Therefore, $TPR_{H_{i}}$ is equal to $FPR_{H_{i}}$ and the point representing such assignment in ROC space lies on the main diagonal, thus having no predictive power.

## Results

A suffix array was constructed for the 5,165 reference sequences in the bacterial taxonomy, and each of the sequencing reads was mapped to these sequences using the GEM-do-index and GEM-mapper tools [50]. GEM-do-index constructs a suffix array from the set of full-length 16S rRNA sequences using the Burrows-Wheeler Transform [57]. Once the sequences have been efficiently indexed, GEM-mapper finds the closest sequences in the suffix array for each of the short sequencing reads in a metagenomic dataset.

**Figure 3:**
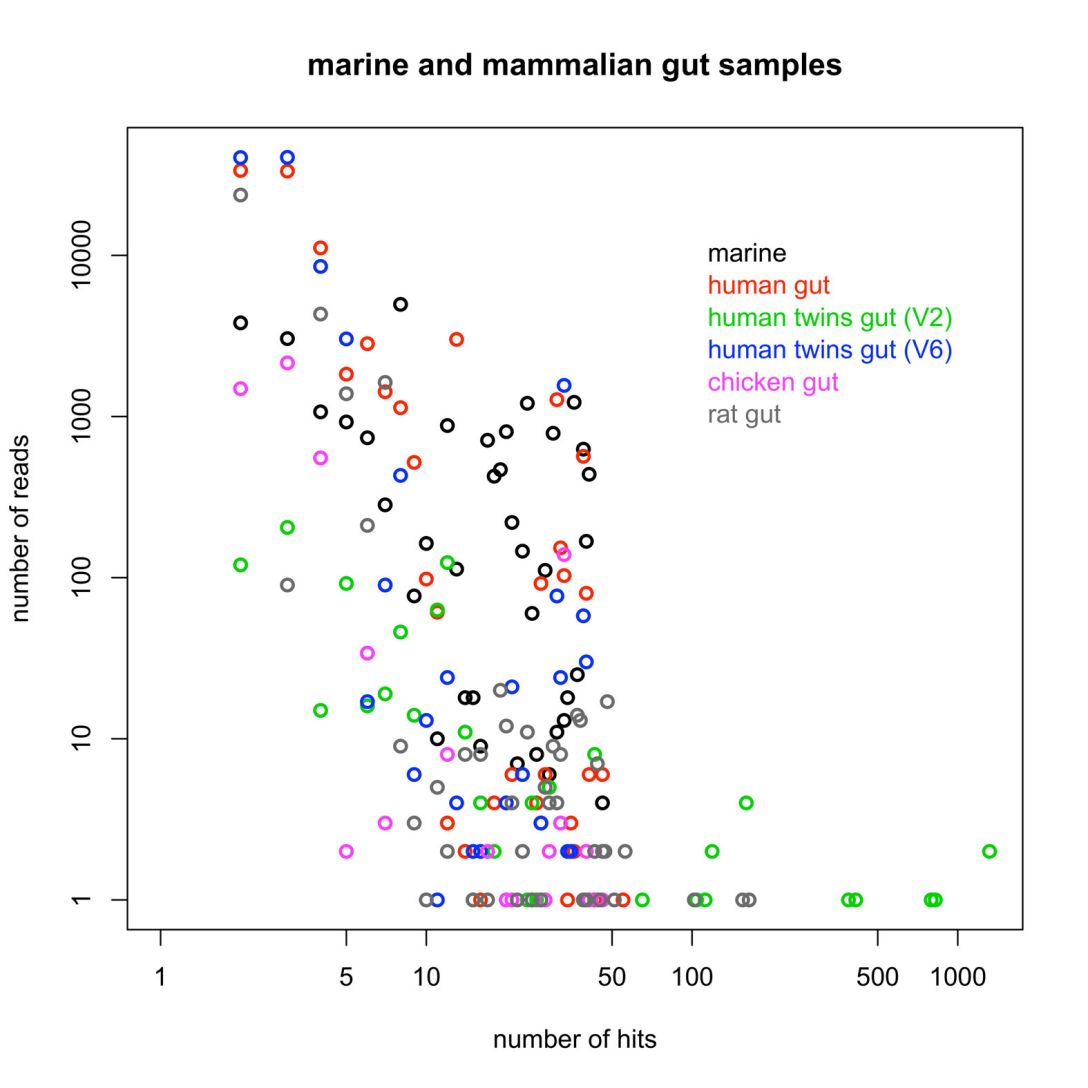
**Distribution of Sequencing Reads**. Distribution of sequencing reads (number of hits and reads) ambiguously assigned with up to 2 mismatches to two or more of the 5,165 sequences in the reference bacterial taxonomy.

**Figure 4:**
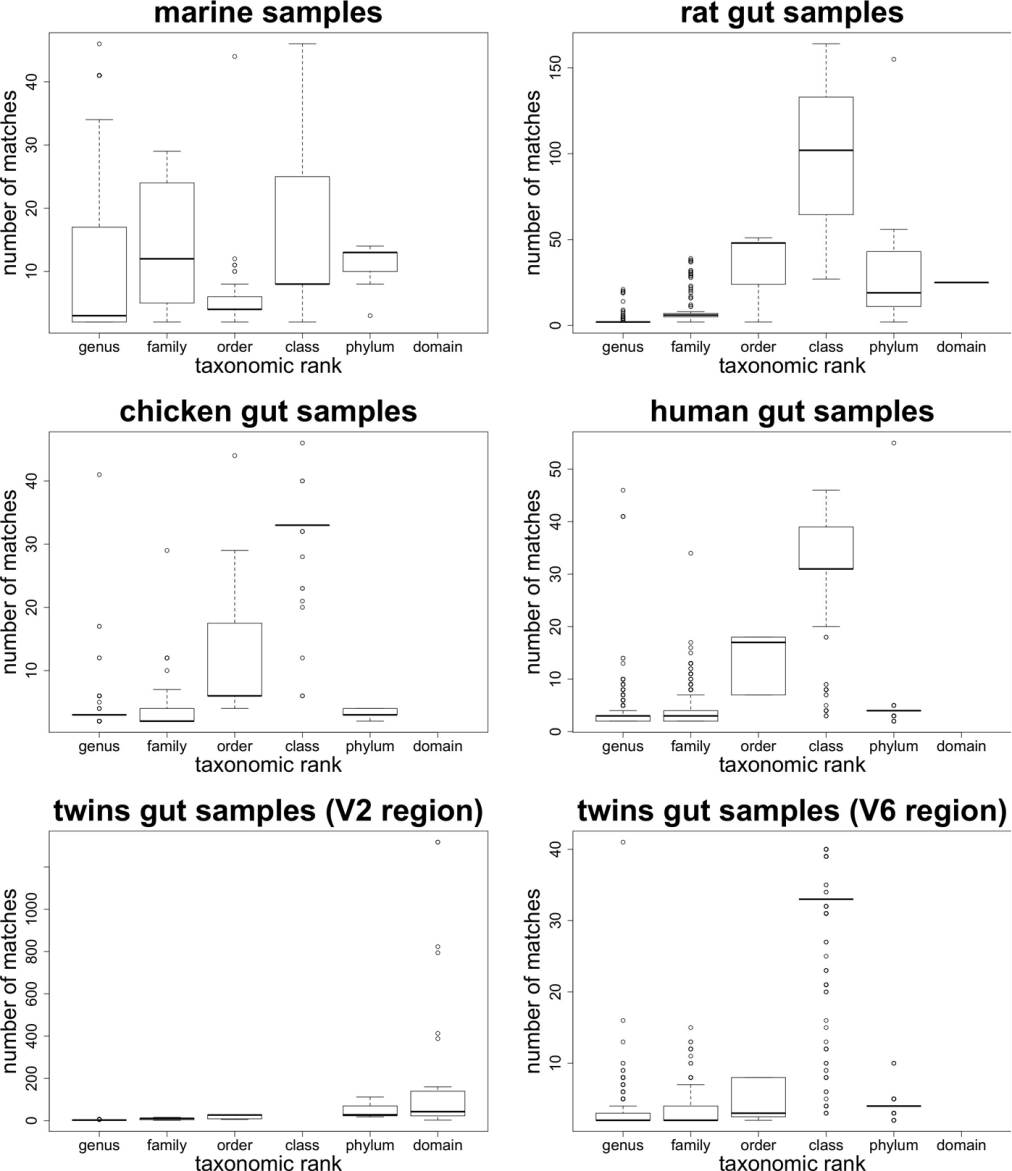
**Distribution of Hits per Taxonomic Rank**. Distribution of the number of hits (species with up to *k *mismatches) in ambiguous reads per taxonomic rank.

Parameters were set to find all matching sequences with up to 2 mismatches, which is about 99% identity for reads of 200 bp. Figure 3 shows the distribution of sequencing reads mapped to more than one sequence in the taxonomy, and Figure 4 shows the distribution of hits per taxonomic rank. Most gut datasets show a distribution of hits that increases with rank up to the class level and then drops (rat, chicken, human, and twins V6 region), while the twins V2 region samples have a disproportionate number of hits at the domain level and the marine dataset does not seem to show a correlation between the number of hits and the taxonomic rank at which reads get assigned.

We performed an alternative mapping using the sequencing reads as BLAST [58] queries against the reference 16S rRNA sequences, defining ambiguous hits as those that could be aligned to more than one species with the same e-value (e-value ≤ 0.001). Table 1 presents a summary of the number of reads that get an assignment with each tool.

**Table 1:** Performance of GEM and BLAST

dataset	GEM-mapper			BLAST		
	
	no hits	1 hit	≥ 2 hits	no hits	1 hit	≥ 2 hits
marine	194,015	4,655	23,621	66,493	8,741	147,057
human	527,727	334,521	91,335	31,881	812,392	109,310
twins V2	990,094	128,649	776	89	1,068,762	50,668
twins V6	523,161	199,782	94,999	36,603	648,975	132,364
chicken	10,442	7,140	4,395	1,548	13,084	7,345
Rat	273,114	27,226	31,509	2	287,971	43,876

Comparison of GEM and BLAST hits for 6 datasets. Two or more hits correspond to ambiguous reads.

Although BLAST hits a larger number of sequencing reads, those reads assigned to one or more species using GEM show a more significative BLAST average e-value than reads with no hits (data not shown). GEM provides a more conservative mapping, discarding those reads that get assigned with lower significance. Allowing for more mismatches with GEM results in a higher number of assigned reads with a higher percentage of ambiguous ones, at the cost of a lower average e-value of the assigned reads (data not shown). The results discussed in this paper, using GEM with up to 2 mismatches, should therefore be considered a conservative estimate.

**Figure 5:**
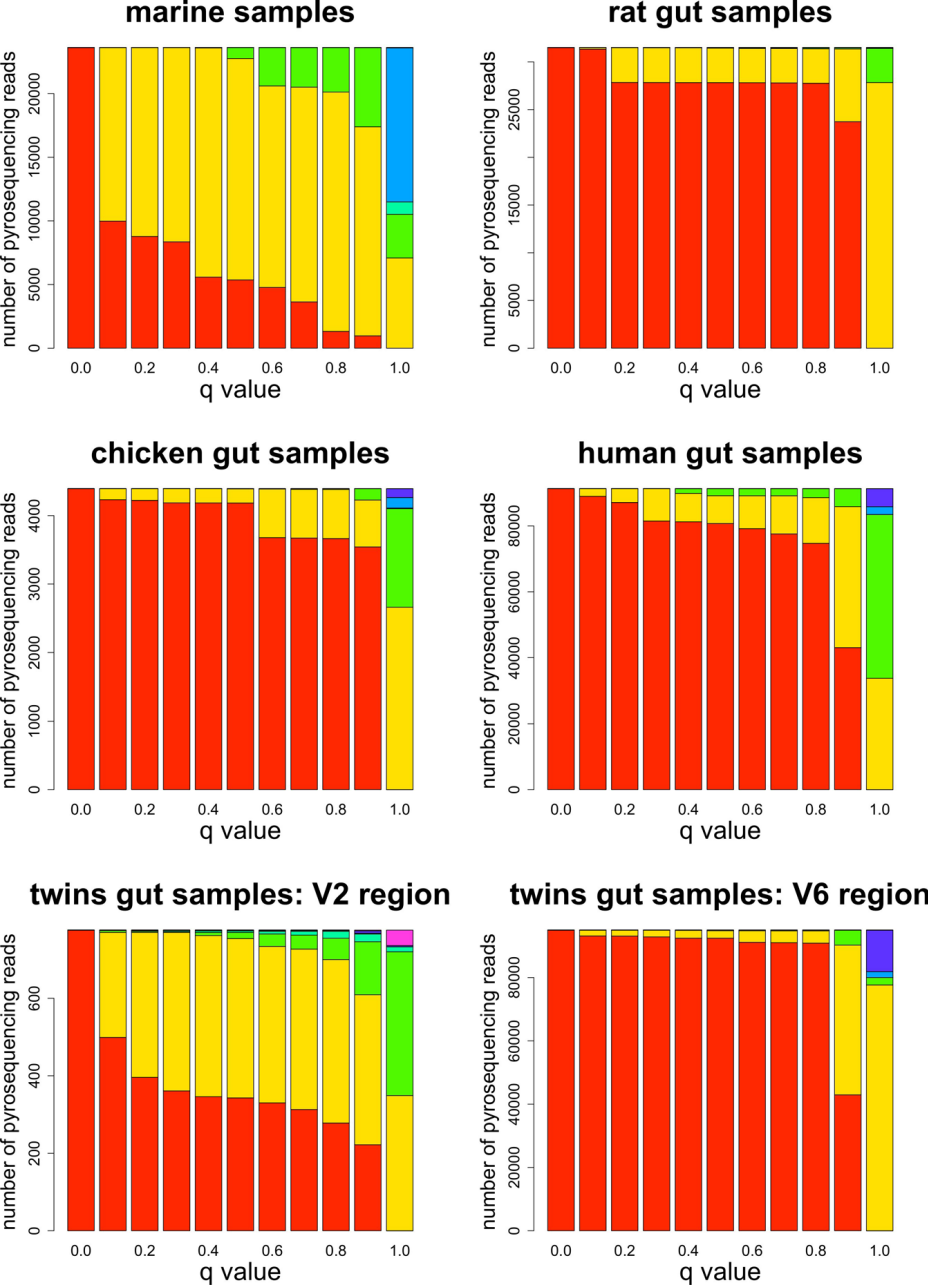
**Distribution of Taxonomic Ranks in Metagenomic Datasets**. Ambiguous reads assigned in the bacterial taxonomy at each taxonomic rank for *q *= 0, ..., 1. Color code: domain: purple; phylum: indigo; class: light blue; order: cyan; family: green; genus: yellow; species: red.

The sequencing reads that matched two or more sequences in the reference bacterial taxonomy were assigned at the taxonomic rank that minimized the penalty score. The distribution of reads assigned at each taxonomic rank is shown in Figure 5 for values of the *q *parameter ranging from 0 to 1. These results show how ambiguous reads can be assigned at the desired taxonomic rank using different values of *q*: low values tend to produce a taxonomic assignment at the genus and species rank, while high values produce a taxonomic assignment at the class, order, and family ranks. The marine dataset seems to have a much higher level of ambiguity, as shown by the large proportion of ambiguous reads that get assigned at the order level for *q *= 1, and by the fact that lower values of *q *still assign many reads above the species level. The twins dataset is particularly interesting in that depending on the sequenced region, the reads are assigned quite differently. With region V2 there is a large percentage assigned above the genus level with *q *= 1, and this percentage is significant even for low values of *q*. The region V6, on the other hand, has most of its ambiguous reads assigned at the genus level when *q *= 1 (although with a notable subset of reads mapped very high, at the class level), but most of the reads get assigned at the species level quickly as we decrease the value of *q*.

### Sequencing Error Bias

Sequencing with 454 suffers mainly from indels in homopolymer runs [30], and such errors can have a significant effect on the final count of bacterial species in a metagenomic sample [48,59]. We analyzed the composition of a bacterial community using a sequencing dataset that included quality scores for each read [48]. Two suffix arrays were constructed: one from the 5,165 high-quality sequences, and one with all the 322,864 16S rRNA sequences found in the Ribosomal Database Project. Each read was then mapped to these taxonomies using the GEM-mapper tool with the parameters described above. The mapping was done with and without the quality scores of the reads, using the error model of 454 sequencing provided by the GEM-mapper tool when incorporating the scores. Table 2 shows the distribution of reads unassigned, assigned with one hit, and assigned with two or more hits (ambiguous reads), with and without quality information.

**Table 2:** Priest Pot Lake sample: Assignment

Priest Pot Lake environmental samples	reference microbial taxonomy					
	
	5,165 type cultures			322,864 sequences		
	
	no hits	1 hit	≥ 2 hits	no hits	1 hit	≥ 2 hits
FASTA	26,458	642	1,261	19,715	991	7,655

FASTQ	26,045	769	1,547	19,182	1,213	7,966

Priest Pot Lake samples: assignment without (FASTA) and with (FASTQ) quality scores for 5,165 type cultures and 322,864 sequences.

Among reads with two or more hits, the maximum number of matches was 82 species (both with and without quality information). Out of 26,458 reads without hits when not using quality information (plain FASTA files), 26,045 also have no hits when incorporating such data (FASTQ files), 158 now have one hit, and 255 have two or more hits. The 642 reads with one hit using FASTA are split into 611 also with a unique hit with FASTQ and 31 with two or more hits. Finally, all 1,261 reads with two or more hits with FASTA also have two or more hits with FASTQ. The distribution of taxonomic ranks with the 5,165 species taxonomy with and without quality scores can be seen in Table 3. Notice how with *q *= 1.0 the presence of a single incorrect species among the hits of a read results in its mapping to the LCA. The use of lower *q *values protects against such erroneous assignments when most of the hits belong to a particular taxonomic group, providing evidence of the read belonging to a taxonomic rank lower than the LCA of all hits.

**Table 3:** Priest Pot Lake sample: Taxonomic Distribution

	leaves				*F*-measure			LCA			
**rank**	**0.0**	**0.1**	**0.2**	**0.3**	**0.4**	**0.5**	**0.6**	**0.7**	**0.8**	**0.9**	**1.0**

domain	0	0	0	0	0	0	0	0	0	0	3
phylum	0	0	0	0	0	0	0	0	0	0	0
class	0	0	0	0	0	0	0	0	0	0	4
order	0	0	0	0	0	1	1	1	1	2	366
family	0	0	1	5	20	47	105	204	232	242	345
genus	0	190	424	455	467	445	561	754	733	727	543
species	1,261	1,071	836	801	774	768	594	302	295	290	0

											
	**leaves**				***F*-measure**			**LCA**			

rank	0.0	0.1	0.2	0.3	0.4	0.5	0.6	0.7	0.8	0.9	1.0

domain	0	0	0	0	0	0	0	0	0	0	27
phylum	0	0	0	0	0	0	0	0	0	0	0
Class	0	0	0	0	0	0	0	0	0	0	15
order	0	0	0	0	1	2	2	2	3	7	419
family	0	2	7	19	43	75	136	266	315	347	393
genus	0	290	529	561	587	568	689	938	902	892	693
species	1,547	1,255	1,011	967	916	902	720	341	327	301	0

Priest Pot Lake samples: taxonomic distribution without (top) and with (bottom) quality scores for 5,165 type cultures. The top row indicates values of the parameter *q *between 0 and 1.

### Assignment Performance

To validate our assignment algorithm we generated six artificial metagenomic datasets using MetaSim, with read lengths 100, 150, and 200 bp for sequences extracted from the whole 16S rRNA sequence or from the V1-V2 hypervariable region only. Out of the original 5,000,000 reads, there were 195,580 (100 bp), 36,462 (150 bp), and 7,637 (200 bp) ambiguous reads when using the whole 16S sequence; and 123,486 (100 bp), 13,147 (150 bp), and 100 (200 bp) ambiguous reads when using the V1-V2 region.

**Figure 6:**
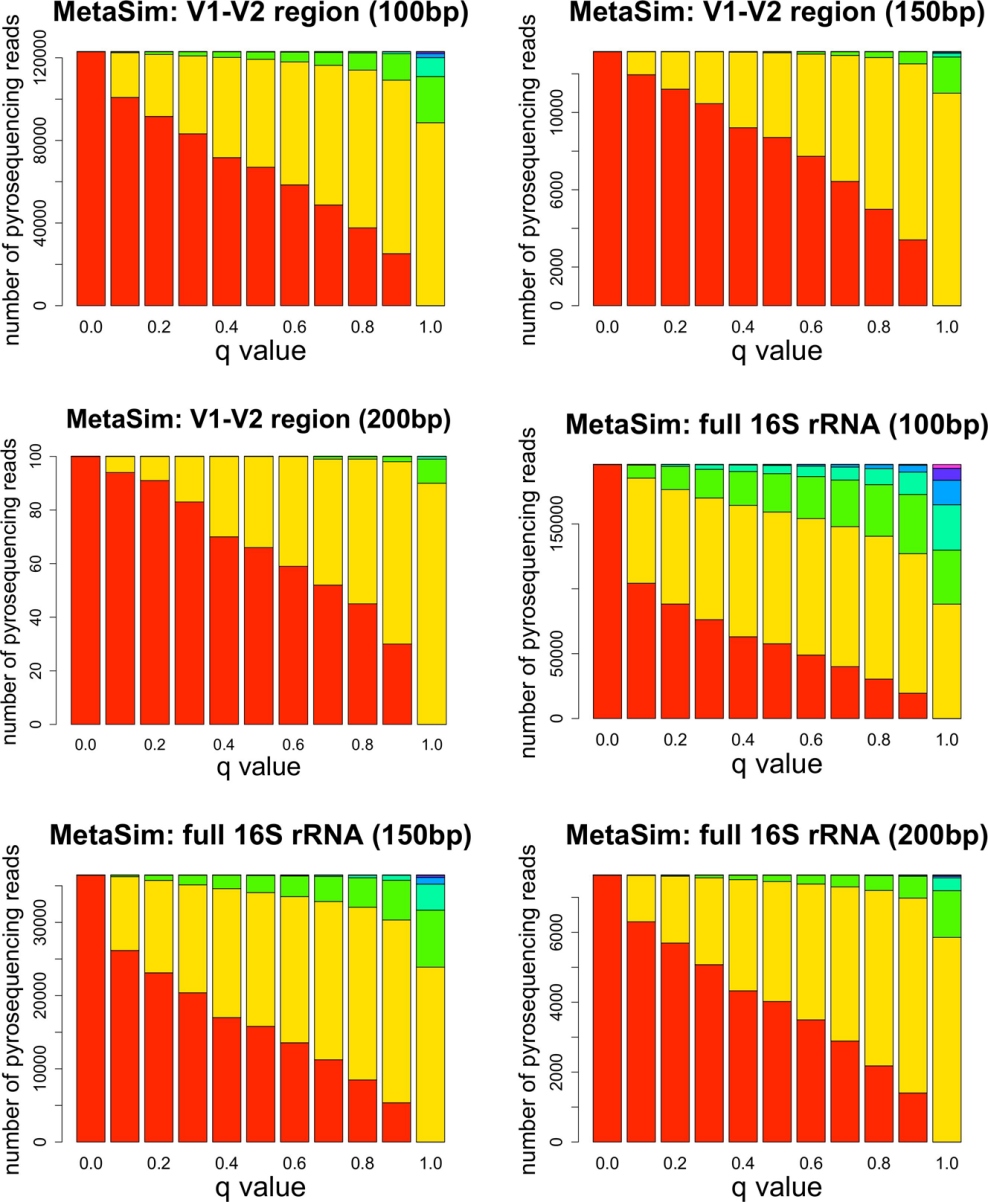
**Distribution of Taxonomic Ranks in Simulated Datasets**. Simulated ambiguous reads assigned in the bacterial taxonomy at each taxonomic rank for *q *= 0, ..., 1. Datasets were constructed from whole 16S rRNA sequence and from the V1-V2 hypervariable region. Color code: domain: purple; phylum: indigo; class: light blue; order: cyan; family: green; genus: yellow; species: red.

Figure 6 shows the distribution of taxonomic ranks for each of the datasets. For 150 and 200 bp, the percentage of ambiguous reads is lower than that observed for experimental datasets, and assignment of these reads to the LCA produces a taxonomic distribution skewed towards lower ranks, with less reads mapped at high taxonomic ranks. Only the dataset generated using 100 bp short reads extracted from complete 16S rRNA gene sequences shows a similar level of ambiguity to that of experimental datasets.

As observed in Table 4 the *q *metric performs better for higher values of this parameter. As the simulated read length decreases, there is a higher proportion of ambiguous reads, more values of *q *result in a positive *G_q _*and the best sum of distances improves: 0.028 (200 bp), 0.048 (150 bp), and 0.096 (100 bp) when using the full-length sequence, and 0.05 (200 bp), 0.015 (150 bp), and 0.036 (100 bp) when using only the V1-V2 region. Assignments with *q *= 0 do not perform particularly well (-0.42, -0.32, and -0.24 for 100, 150, and 250 bp extracted from the full-length 16S, and -0.23, -0.16, and -0.08 for 100, 150, and 250 bp obtained from the V1-V2 region), indicating that in most cases the true hit *H *would not be in the chosen subtree if we choose a single leaf out of all possible hits. This is more so as the read length decreases and ambiguous reads are mapped at higher taxonomic ranks, resulting in a larger number of possible hits (*M_i_*) and a lower probability of choosing the true hit (*M_i_*,*_j_/M_i_*).

**Table 4:** Validation of Results: Gq

metric	length	∑ *D_i_*	0.0	0.1	0.2	0.3	0.4	0.5	0.6	0.7	0.8	0.9	1.0
*G_q _*[16S]	100	0.096	-0.42	-0.13	-0.06	-0.01	0.01	0.03	0.05	0.07	0.08	0.09	0.00
	150	0.048	-0.32	-0.14	-0.09	-0.05	-0.02	-0.00	0.01	0.02	0.03	0.04	0.00
	200	0.028	-0.24	-0.13	-0.09	-0.06	-0.04	-0.02	-0.01	0.00	0.01	0.03	0.00

*G_q_* [V1-V2]	100	0.036	-0.23	-0.12	-0.08	-0.04	-0.03	-0.01	0.00	0.01	0.02	0.03	0.00
	150	0.015	-0.16	-0.11	-0.08	-0.05	-0.04	-0.02	-0.01	-0.00	0.00	0.01	0.00
	200	0.005	-0.09	-0.05	-0.01	-0.00	-0.00	-0.00	-0.00	0.00	0.00	0.00	0.00

Sum of distances *G_q _*for each value of *q *in simulations using the full-length 16S rRNA gene sequence (top) and the V1-V2 hypervariable region (bottom) for reads of length 100, 150, and 200 bp. The column ∑ *D_i _*indicates the best sum of distances achieved.

The expected distance metric *E*(*D_i_*) maps half of the reads to the LCA, and the rest get evenly distributed among all *q *values, as seen in Table 5. As with the *q *metric, more ambiguous datasets produce better results, and the sum of distances gradually increases as well: 0.073 (200 bp), 0.086 (150 bp), and 0.125 (100 bp) when using the full-length sequence, and 0.031 (200 bp), 0.058 (150 bp), and 0.077 (100 bp) when using the V1-V2 region. The best sum of distances is always larger with *E*(*D_i_*) than with *G_q_*, indicating that a combination of values of *q *is a better predictor than assigning all reads using a unique value.

**Table 5:** Validation of Results: Assignments.

metric	length	*∑ D_i_*	0.0	0.1	0.2	0.3	0.4	0.5	0.6	0.7	0.8	0.9	1.0
*E*(*D_i_*) [16S]	100	0.125	2.3%	3.9%	4.5%	5.1%	5.0%	5.4%	5.7%	6.2%	7.0%	9.6%	45.0%
	150	0.080	3.5%	4.5%	4.9%	5.4%	4.9%	5.2%	5.2%	5.2%	5.2%	5.4%	50.0%
	200	0.070	4.3%	4.8%	5.1%	5.5%	4.8%	5.2%	5.1%	4.8%	4.4%	4.1%	51.4%

*E*(*D_i_*) [V1-V2]	100	0.077	4.6%	5.2%	5.6%	5.9%	5.3%	5.5%	5.3%	5.0%	4.9%	5.0%	47.1%
	150	0.056	5.7%	6.0%	6.1%	6.3%	5.5%	5.7%	5.5%	4.7%	4.1%	3.4%	46.5%
	200	0.023	7.1%	7.3%	7.3%	7.4%	6.2%	6.2%	5.4%	4.5%	4.0%	3.0%	41.0%

Percentage of reads assigned at the node selected for each value of *q *when maximizing *E*(*D_i_*) in simulations using the full-length 16S rRNA sequence (top) and the V1-V2 hypervariable region (bottom) for reads of length 100, 150, and 200 bp. The column ∑ *D_i _*indicates the best sum of distances achieved.

Table 6 shows precision and recall values for the *q *metric in the six artificial datasets. Precision decreases and recall increases with higher values of this parameter, as expected. Moreover, reads extracted from full-length sequences tend to have more matches than reads extracted from the V1-V2 region and thus, precision values are higher for reads extracted from the V1-V2 region than for reads extracted from full-length sequences (the latter contain more false positives), and recall values tend to be higher for reads extracted from full-length sequences than for reads extracted from the V1-V2 region (the latter contain more false negatives).

**Table 6:** Validation of Results: Precision and Recall.

	full 16S rRNA						V1-V2 region					
	100 bp		150 bp		200 bp		100 bp		150 bp		200 bp	
***q***	***p***	***r***	***p***	***r***	***p***	***r***	***p***	***r***	***p***	***r***	***p***	***r***

0.0	0.1827	0.1827	0.2503	0.2503	0.3038	0.3038	0.3085	0.3085	0.3553	0.3553	0.4021	0.4021
0.1	0.1820	0.4416	0.2480	0.4296	0.3003	0.4252	0.3059	0.4246	0.3530	0.4194	0.4085	0.4536
0.2	0.1791	0.5165	0.2448	0.4920	0.2954	0.4793	0.3026	0.4819	0.3502	0.4644	0.4119	0.4948
0.3	0.1742	0.5773	0.2401	0.5551	0.2863	0.5367	0.2960	0.5319	0.3453	0.5071	0.4012	0.5464
0.4	0.1677	0.6343	0.2314	0.6205	0.2752	0.5996	0.2873	0.5924	0.3394	0.5688	0.3741	0.6020
0.5	0.1633	0.6653	0.2262	0.6500	0.2707	0.6322	0.2816	0.6205	0.3326	0.5939	0.3558	0.6224
0.6	0.1548	0.7054	0.2147	0.6938	0.2586	0.6779	0.2687	0.6666	0.3198	0.6417	0.3427	0.6633
0.7	0.1436	0.7483	0.2001	0.7393	0.2399	0.7295	0.2476	0.7156	0.2924	0.7000	0.3343	0.7143
0.8	0.1284	0.7941	0.1767	0.7898	0.2123	0.7909	0.2189	0.7718	0.2571	0.7620	0.3108	0.7551
0.9	0.1081	0.8519	0.1465	0.8506	0.1735	0.8544	0.1815	0.8383	0.2146	0.8366	0.2480	0.8367
1.0	0.0532	0.9628	0.0719	0.9700	0.0818	0.9788	0.0807	0.9726	0.0893	0.9788	0.1084	0.9700

Precision (*p*) and recall (*r*) for each value of *q *in simulations using the full-length 16S rRNA sequence and the V1-V2 hypervariable region (right) for reads of 100, 150, and 200 bp.

## Discussion

Comparison of results between GEM and BLAST show that, although BLAST can map more reads, there is still a large number of reads that either cannot be assigned to any species in the taxonomy, or can be assigned to more than one species. GEM provides a more conservative assignment, with assigned reads having a more significant e-value in BLAST. The combined speed of GEM in assigning species to each read and of our algorithm in mapping ambiguous reads to the taxonomic rank minimizing the penalty score provides a useful tool to quickly test hypotheses about microbial communities.

Ambiguous reads are assigned to different taxonomic ranks depending on the value of *q*, as shown in Figure 5 Figure 6 and Table 3. As the value of *q *increases, more reads get assigned at higher taxonomic ranks, with clearly different distributions between the extreme values *q *= 0 and *q *= 1. Ambiguous and unassigned reads could either belong to species not present in the taxonomy or be artifacts due to errors in the experimental process. Bacterial taxonomies are biased towards cultivable species, but the human gut and oceanic environments are known to harbor many rare, uncultivable bacteria [7,20]. Even if a large number of reads come from unknown species, most of them have a small number of hits (as seen in Figure 3) and would only introduce a moderate amount of error. Reads with a large number of hits, such as some of the reads coming from the twins V2 region dataset, might be chimeric sequences product of the PCR amplification step [60], or reads belonging to species from yet to be identified taxonomic groups. Reads not uniquely mapped can also be caused by sequencing errors, most commonly homopolymer indels when using 454 sequencing [61]. We would expect to observe differences in the distributions of homopolymers between the sets of ambiguous and unambiguous reads, but we could not find a significantly higher number of homopolymers for any of these sets across all our datasets (data not shown). Analysis of the distributions of homopolymer lengths versus the number of homopolymers per read was also inconclusive, and we could not clearly differentiate the distributions in ambiguous and unambiguous reads. A BLAST search using reads with no hits as both query and database showed that the vast majority can be aligned with high significance (often perfect matches) to other no-hit reads, indicating that either the same type of sequencing error occurred frequently or that these reads cannot be mapped because the corresponding 16S rRNA sequence is not in the taxonomy. On the other hand, incorporating read quality information reduces the number of unassigned reads but increases the number of ambiguous reads more significantly than that of uniquely assigned reads (see Table 3), stressing the importance of an assignment of ambiguous reads that minimizes bias in the estimation of diversity.

Artificial datasets produced through simulations contained a lower percentage of ambiguous reads when compared to experimental datasets for similar read lengths, and most ambiguous reads were assigned at lower taxonomic ranks. The simulations using the V1-V2 hypervariable region to extract short reads were expected to mimic experimental conditions more closely, but they showed even less ambiguity than simulations using the full-length 16S rRNA sequence to obtain the short reads. The results described in Section "Assignment Performance", therefore, represent a low estimate of performance, and datasets with a higher proportion of ambiguous reads would further benefit from our assignment algorithm. Tables 4 and 5, in fact, show how simulations with a higher proportion of ambiguous reads benefit more from our taxonomic mapping algorithm. Although the sequencing error models utilized in the simulations probably differ slightly from the actual errors, we believe the increased ambiguity observed in the experimental datasets is due to a combination of several factors: sequencing errors, PCR artifacts, and incomplete taxonomic information for some of the species present in the samples. Selecting a unique *q *value to assign ambiguous reads based on these simulations might therefore produce biased results and, until more realistic simulations can be performed, we suggest the use of the estimated distance metric instead, which does not require an estimation of an optimal value for *q*. It should be noticed that, both in the *q *metric and in the expected distance metric, the distances are relevant to determine whether the assignment to the LCA can be outperformed, but the true measure of significance is given by the observed differences in the distribution of taxonomic ranks, as seen in Figure 5 and Figure 6.

## Conclusions

In this paper, we have introduced a new method for the taxonomic assignment of ambiguous sequencing reads based on a generalization of the *F*-measure that minimizes a penalty score combining the precision and recall of the mapping. There is, to the best of our knowledge, no other taxonomic assignment method concerning precision and recall, apart of the assignment to the LCA. By using a suffix array representation of the sequences in the leaves of the taxonomy and preprocessing the taxonomy for fast search of relevant nodes, our assignment algorithm can work in time linear in the number of sequences matching a read. Our algorithm can analyze large metagenomic datasets even on a small PC. For instance, on a MacBook Pro with 8GB of memory, the analysis of the Priest Pot Lake dataset takes approximately 30 minutes for GEM to analyze (up to 7 mismatches), and another 30 minutes to assign ambiguous reads. The use of a single parameter to control whether precision or recall should be prioritized results in assignments with clearly different distributions of taxonomic ranks. The assignment strategy of sequencing reads introduced in this paper is therefore both a versatile and a quick method to study bacterial communities.

The study of six different datasets of environmental and gut samples shows that the composition and diversity of bacterial species observed in a sample can vary significantly depending on whether ambiguous or unambiguous reads are used, and on the particular value of the *q *parameter. Results with a dataset where read quality information is provided shows that the number of ambiguous reads increases when such information is used, making our algorithm more relevant. Validation of the assignment schema in an artificial dataset shows that a combination of different *q *values produces the most accurate results. The fact that a unique set of sequencing reads can produce very different distributions depending on how the large number of ambiguous reads are assigned has deep implications for metagenomic studies in general, and in particular for those trying to correlate bacterial composition with disease states. A more accurate characterization of these reads can therefore provide a better understanding of the microbial diversity around and within us.

## Availability

The software and data sets are available under the GNU GPL at the supplementary material web page http://www.lsi.upc.edu/~valiente/tango/.

## Authors' contributions

All authors conceived the method, prepared the manuscript, contributed to the discussion, and have approved the final manuscript. GV implemented the software. JC also implemented part of the software.
